# Dielectric barrier discharge plasma catalysis as an alternative approach for the synthesis of ammonia: a review

**DOI:** 10.1039/d3ra05580a

**Published:** 2023-09-25

**Authors:** Hamideh Hosseini

**Affiliations:** a Chemistry and Chemical Engineering Research Center of Iran (CCERCI) PO Box 14335-186 Teheran Iran h.hosseini1173@yahoo.com

## Abstract

Numerous researchers have attempted to provide mild reactions and environmentally-friendly methods for NH_3_ synthesis. Research on non-thermal plasma-assisted ammonia synthesis, notably the atmospheric-pressure nonthermal plasma synthesis of ammonia over catalysts, has recently gained attention in the academic literature. Since non-thermal plasma technology circumvents the existing crises and harsh conditions of the Haber–Bosch process, it can be considered as a promising alternative for clean synthesis of ammonia. Non-thermal dielectric barrier discharge (DBD) plasma has been extensively employed in the synthesis of ammonia due to its particular advantages such as the simple construction of DBD reactors, atmospheric operation at ambient temperature, and low cost. The combination of this plasma and catalytic materials can remarkably affect ammonia formation, energy efficiency, and the generation of by-products. The present article reviews plasma-catalysis ammonia synthesis in a dielectric barrier discharge reactor and the parameters affecting this synthesis system. The proposed mechanisms of ammonia production by this plasma catalysis system are discussed as well.

## Abstract 2

We discuss the synthesis of ammonia over catalysts using dielectric barrier discharge plasma as a promising alternative approach.

## Graphical abstract

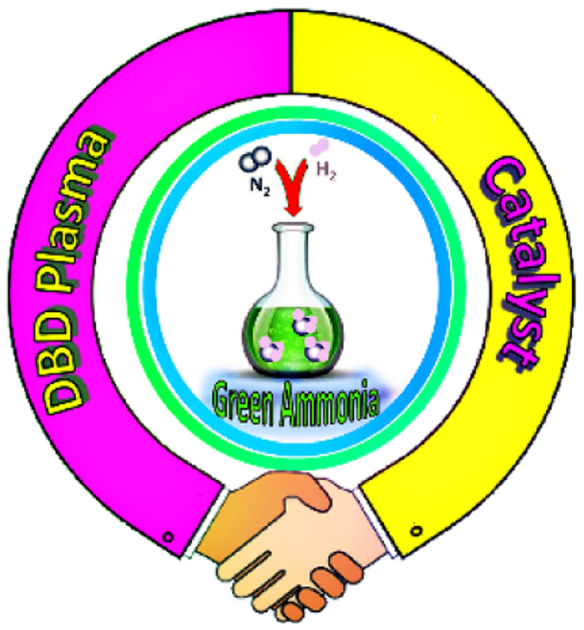

## 1. Introduction

### 1.1. Ammonia: importance and application

**Fig. 1:**
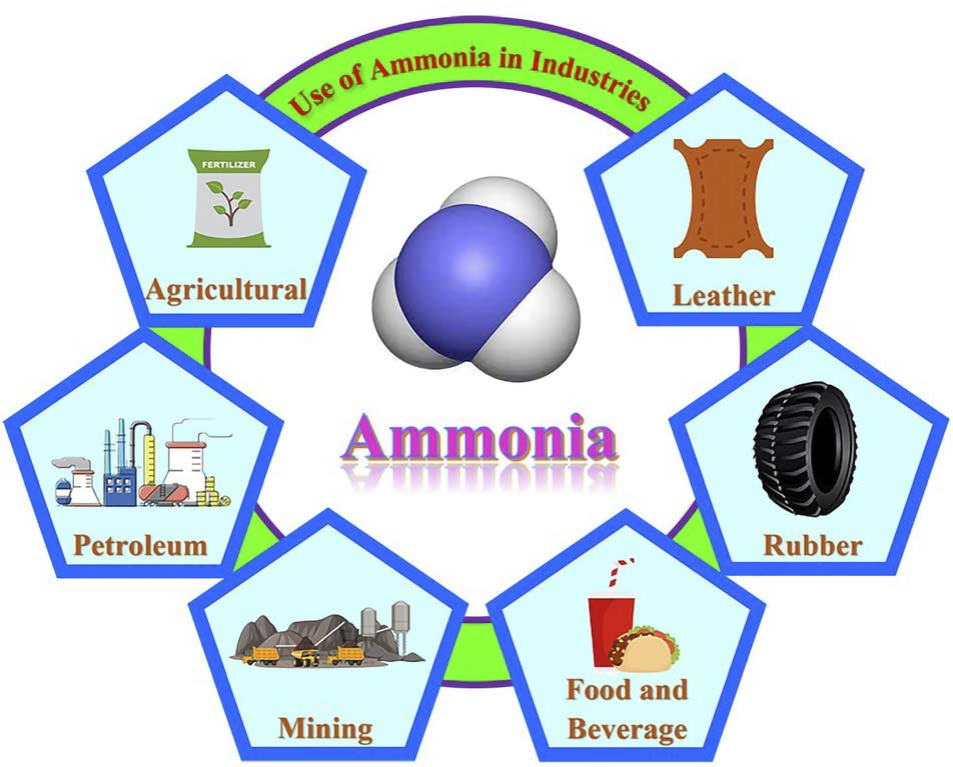
Use of ammonia in different industries.

Ammonia (NH_3_), as a nitrogenous valuable source, is used in the manufacture of plastics and fibers, pharmaceuticals, explosives, chemicals, ammonium fertilizers such as carbamide ammonium nitrate and ammonium bicarbonate, *etc.*^1^ A number of the applications of ammonia in industries are shown in Fig. 1. Ammonia is also employed as an indirect hydrogen storage material.^2^ In recent years, the surface functionalization of carbon nanotubes *via* plasma treatments has been carried out by means of ammonia.^3^ The formation of organic compounds containing C≡N bonds is another achievement of the use of ammonia in the plasma system.^4^ As compared to other nitrogenous compounds, this compound is produced on a very large molar scale in the industry. The global production of ammonia reported was about 150 million metric tons in 2022.^5^ Ammonia, in fact, has been known worldwide as a chemical for more than two centuries. Johann Jacob Wepfer was indeed the first researcher to detect ammonia by distillation of putrefied wine yeast in 1679, but Joseph Priestley became known as the discoverer of gaseous ammonia in 1774.^1^ A few years later, in 1785, ammonia was identified by Claude Louis Berthollet as a compound synthesized from N_2_ and H_2_,^6^ while the preparation of ammonia from its elements was first carried out by Humphry Davy in 1807.^7^ Until 1900, many scientists and researchers such as William Donkin, Oliver Lodge, Ramsay and Young and others worked on ammonia and obtained remarkable results and achievements.^8–10^ In line with this research, Fritz Haber synthesized ammonia from hydrogen and nitrogen in desirable amounts in 1909,^1^ which will be discussed in more detail below.

### 1.2. Ammonia synthesis

Several approaches have been reported in various literatures that were employed to prepare ammonia such as Haber–Bosch process, green Haber–Bosch, electrochemical synthesis, photochemical synthesis, chemical looping process, and plasma-catalysis synthesis which are briefly described in the following sections.

### 1.3. Haber–Bosch process

**Fig. 2:**
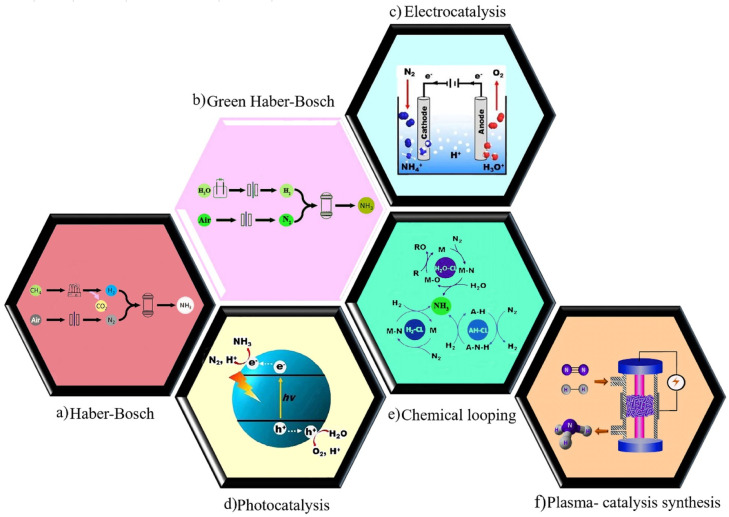
Schematic illustration of (a) Haber–Bosch process, (b) the green Haber–Bosch process. Reproduced from ref. 103 with permission from Elsevier, copyright 2021. (c) Electrochemical, (d) photochemical, (e) chemical looping. Reproduced from ref. 24 with permission from Elsevier, copyright 2019. (f) Plasma-catalysis synthesis. Reproduced from ref. 87 with permission from Wiley-VCH, copyright 2017.

In the early 20th century, Fritz Haber and Carl Bosch developed ammonia synthesis using the method of directly synthesizing ammonia from hydrogen and nitrogen with a metal catalyst called Haber–Bosch (HB) process as shown in Fig. 2a. Ammonia synthesis is industrially carried out at high pressure (150–300 bar) and temperature (450–600 °C). Meanwhile, the production of ammonia is reversible and is considered an exothermic reaction. So, by decreasing the temperature according to the Le Chatelier principle, the balance can be shifted towards more ammonia production. On the other hand, lowering the temperature causes the equilibrium and the ammonia production rate to be very low. Therefore, in order to further increase the equilibrium rate and thus the rate of ammonia production, this reaction is performed in the presence of a highly active catalyst. Accordingly, the use of a catalyst in this synthesis is essential to accelerate the reaction.^11–13^ Also, it has been estimated that this process produces more than 300 million tons of CO_2_ annually and consumes up to 2% of the world's energy.^14^ Therefore, it seems logical that the HB process should be replaced by a more environmentally friendly and economically efficient process.

### 1.4. Green Haber–Bosch

One of the effective alternatives for the production of ammonia in a clean, green, and sustainable way is the electrocatalytic synthesis of ammonia from nitrogen and water under mild reaction conditions using renewable electricity.^15^ The utilization of water as the hydrogen source in this approach can be a noteworthy advantage compared with HB process as shown in Fig. 2b. Similar to the Haber–Bosch process, ammonia is produced under high pressure and temperature in this approach.

### 1.5. Electrochemical synthesis

The electrochemical synthesis of ammonia has been introduced as an appealing alternative to the Haber–Bosch process due to its mild operating conditions, lack of carbon dioxide emission, ability to store renewable energies in chemical bonds, and possibility for distributed ammonia production. This synthesis is possible *via* nitrogen reduction reaction (NRR) or nitrogenous pollutants reduction (Fig. 2c). However, this approach is still in its infant stage and is faced with numerous obstacles.^16^ Electrochemical synthesis of ammonia has been extensively discussed in some publications.^17–21^

### 1.6. Photochemical synthesis

An environmentally-friendly approach to synthesize ammonia from N_2_ and water driven by solar energy is photocatalytic synthesis of ammonia.^22^ Despite significant photocatalyst development so far, further advances are required to make practical NH_3_ production possible.^23^ In general, photochemical NRR entails the absorption of light to produce photoexcited charge carriers, separation and migration of the electron–hole pairs to the reactive sites of the surface, and reaction of photo-induced electrons with nitrogen to produce ammonia with an uptake of water-derived protons.^24^ The schematics illustration of this approach is shown in Fig. 2d. Here, no further details will be discussed as the focus of this review is plasma-catalytic synthesis.

### 1.7. Chemical looping process

Chemical looping for the production of ammonia has received a large amount of interest. In this process, the first step involves the contact of N_2_ with a solid-state transition metal to generate a nitride (activation) and, in the second step, NH_3_ is obtained by contacting the nitride with steam or hydrogen. Among the benefits of chemical looping, in addition to the ability to independently control the conditions for N_2_ activation and product harvest, is the ability to operate at atmospheric pressure for nitrogen activation (Fig. 2e).^25^

### 1.8. Plasma-catalysis synthesis

The plasma catalysis process involves integrating plasma and catalysts in order to attain reactant conversions and product selectivities that are not possible with either plasma or catalyst alone. Although plasma-catalytic ammonia production has been known since the early 1900s, the optimization of this reaction is currently the focus of a lot of ongoing research (Fig. 2f).^26^

Despite the valuable achievements that have been made so far for mild-condition NH_3_ synthesis by electrochemical, photochemical, chemical looping, and plasma catalysis processes, there are still challenges and limitations in each of the mentioned approaches that researchers are facing. Therefore, more studies need to be carried out to solve the existing challenges in these approaches.

This review aims to further contribute towards the understanding of catalyzed ammonia synthesis in the DBD reactor under mild conditions, which has recently attracted the attention of researchers. The basic concepts of plasma-catalysis and the classification of plasma-assisted catalysis for chemical reactions are discussed in Section 2. The production of ammonia in the plasma catalysis system and the factors affecting this synthetic process are discussed in Section 3.

## 2. Plasma-catalysis

As mentioned above, the plasma catalysis process is one of the alternative approaches for ammonia production. In order to better understand this process, some fundamental concepts in this field are discussed.

### 2.1. Plasma

Plasma as the fourth state of matter, comprising 99.9% of the visible universe, contains high-energy electrons, free radicals, active ions, and excited species. This term was first proposed by Langmuir in 1928. Accordingly, plasma is classified into two categories of high and low temperature plasma based on the internal temperature of electrons. There are two types of low temperature plasma: thermal and non-thermal plasma (NTP). NTPs can be divided into atmospheric pressure plasma and low pressure plasma.

As the temperature in non-thermal plasmas is close to ambient temperature, these plasmas are suitable for most chemical reactions. Glow discharge, radiofrequency plasma (RF), dielectric barrier discharge, and atmospheric pressure plasma jet are non-thermal plasmas or cold plasmas that have the most application compared to other plasmas.^27,28^ Accordingly, NTPs can be considered an alternative approach to the synthesis of chemicals, notably those whose synthesis requires the use of high temperatures and/or pressures or other harsh conditions.^4,29^

### 2.2. Dielectric barrier discharge

**Fig. 3:**
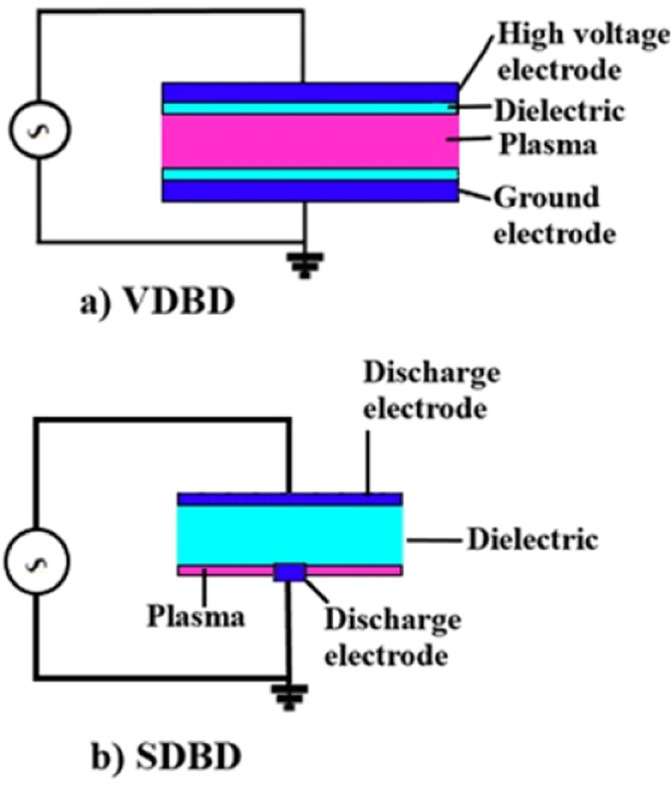
Two types of dielectric barrier discharges: (a) volume dielectric barrier discharge (b) surface dielectric barrier discharge. Reproduced from ref. 31 with permission from Wiley-VCH, copyright 2020.

In 1857, Siemens utilized dielectric barrier discharge (DBD) plasma for the generation of ozone.^30^ Since that time many types of DBD designs and geometries were made and used for different applications. DBDs, also known as silent discharges, are created using an insulating (dielectric) material to generate self-pulsing plasma between the electrodes.^3^ Based on the configuration of the setup, there are two main categories for DBDs including volume dielectric barrier discharge (VDBD) and surface dielectric barrier discharge (SDBD) as shown in Fig. 3. In VDBD, plasma is generated in the space between two electrodes which includes a dielectric and discharge gap and whilst in SDBD the space between the electrodes is completely filled by a dielectric and plasma is created on the surface of the dielectric.^31^ There are various configurations of SDBD and VDBD geometries such as symmetric VDBD with two dielectric barriers, VDBD with a floating dielectric barrier, asymmetric VDBD, symmetric single-sided SDBD, asymmetric SDBD, coplanar SDBD, symmetric double-sided SDBD, cylindrical VDBD, packed-bed DBD which can be found in ref. 31.

### 2.3. Catalyst

Catalysts are substances accelerating the rate of chemical reactions by reducing the activation energy or changing the reaction mechanism without themselves being consumed in the process. In other words, the catalyst and a reactant react to form chemical intermediates and then the reaction of intermediates with each other or with another reactant leads to the formation of the final desired product. Catalysts can be solid, liquid, or gases. Homogeneous and heterogeneous catalysts are two basic types of catalysts. Currently, most chemical reactions are carried out through catalytic processes, especially heterogeneous catalysis. Catalyst synthesis requires specialized facilities and can be a complex process.^32^

### 2.4. Plasma catalysis

Plasma-catalysis is a combination of plasma and catalytic materials which are present in the numerous plasma processes. Plasma-catalysis with its various applications such as waste water treatment, material treatment, volatile organic compounds (VOC), indoor air cleaning, methanation, H_2_ formation, CO_2_ reduction, and the synthesis of NH_3_ has attracted a lot of attention among the researchers, especially the chemists.^33^ As plasma is an environment full of active species, including energetic electrons, ions, radicals, and excited molecules and neutrals, it is difficult to perform chemical reactions with high selectivity. In order to achieve increased selectivity of target products and improved energy efficiency in chemical reactions, it is necessary to use a plasma catalysis system, so the presence of plasma and catalyst together can be effective for performing many chemical reactions. To date, a large number of catalysts have been introduced and used for the plasma-assisted catalytic system in chemical reactions. According to the published articles in this field, oxide supports (TiO_2_, Al_2_O_3_, and SiO_2_)^34–38^ and different zeolites,^39,40^ supported oxides and mixed oxides (intimate mixed oxides and perovskites),^41–44^ and metal catalysts such as embedded nanoparticles, supported metals, and metal wires are reported more than other catalysts in plasma-assisted catalytic reactions.^45,46^ Generally, the catalysts applied in plasma reactors are in the form of tablets (pills), granules, extrudates, pellets, and foams.^47^ These structures affect the performance of catalysts in plasma-assisted catalytic processes.

### 2.5. The classification of plasma-assisted catalysis for chemical reactions

Four plasma-catalysis systems, single-, two-, multi-stage, as well as cycled system, are considered for the plasma combined with the catalyst, depending on the number of catalyst beds and the position of the catalyst. Single-stage system, also called in-plasma catalysis (IPC), is a configuration where the catalyst is packed in the discharge zone (a). Therefore, the catalyst and plasma are in direct contact with each other. In a two-stage configuration also called post-plasma catalysis (PPC), the catalyst is after the discharge zone (b). In this case, plasma and catalysis cannot interact directly with each other. Additionally, it is possible to combine catalysts with different functions in a multi-stage configuration to obtain the desired and expected plasma treatment (c). This configuration can be an interesting and applicable option in the future notably in industry. Lastly, the cycled system (d) involves two steps: adsorption and plasma decomposition of the contaminants adhering to the surface.^48,49^ The first two configurations, IPC and PPC which are more common in most reactions, are discussed below.

### 2.6. In-plasma catalyst (IPC)

**Fig. 4:**
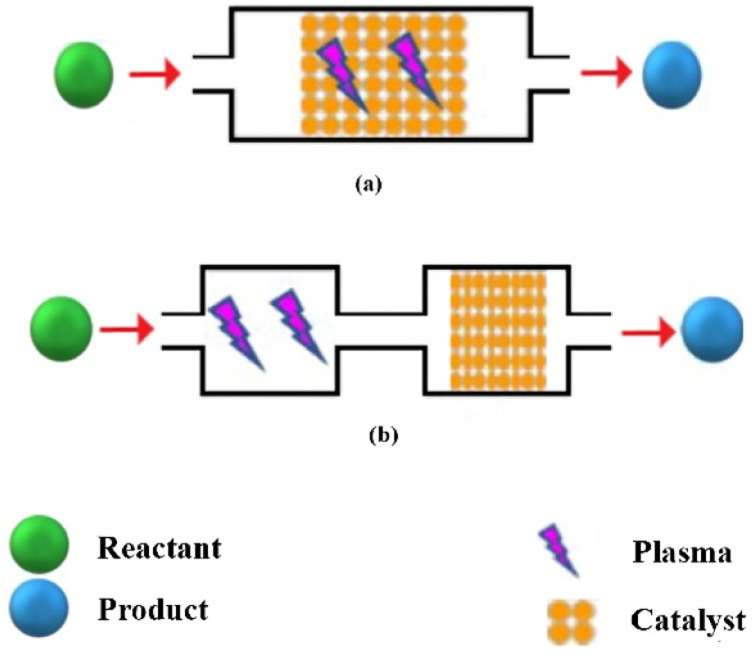
Two types of plasma-catalysis reactors (a) single-stage system (b) two-stage system.

Plasma-assisted catalysis uses the energy obtained from the excitation of the plasma to activate species either in the gas phase or on the catalyst surface. As shown in Fig. 4a, in IPC configuration, catalysts are placed in the discharge region.^49^ So, the catalyst interacts directly with the plasma and with the reaction products, thereby affecting the chemical nature of the process.^50^ In this configuration, active species such as excited-state atoms and molecules, reactive radicals, photons, and electrons generated by plasma are generally short-lived. The plasma in this one-stage arrangement may be responsible for preparing or modifying the catalyst surface.^49^

### 2.7. Post-plasma catalyst (PPC)

In a two-stage arrangement, the catalyst is located downstream of the plasma and is only exposed to species that are released from the plasma (Fig. 4b). Normally, these species are the end-products of the gas phase plasma processing or long-lived intermediates and maybe vibrationally excited species as well.^49^ Methane partial oxidation to methanol (MPOM) is one of the reactions that is usually performed with the PPC configuration. It seems that the use of the catalyst in PPC configuration in this reaction has some advantages such as its high resistance to carbon deposition and its long-time stability in extended MPOM reactions.^51^

## 3. Plasma-catalysis ammonia synthesis in DBD reactors

**Fig. 5:**
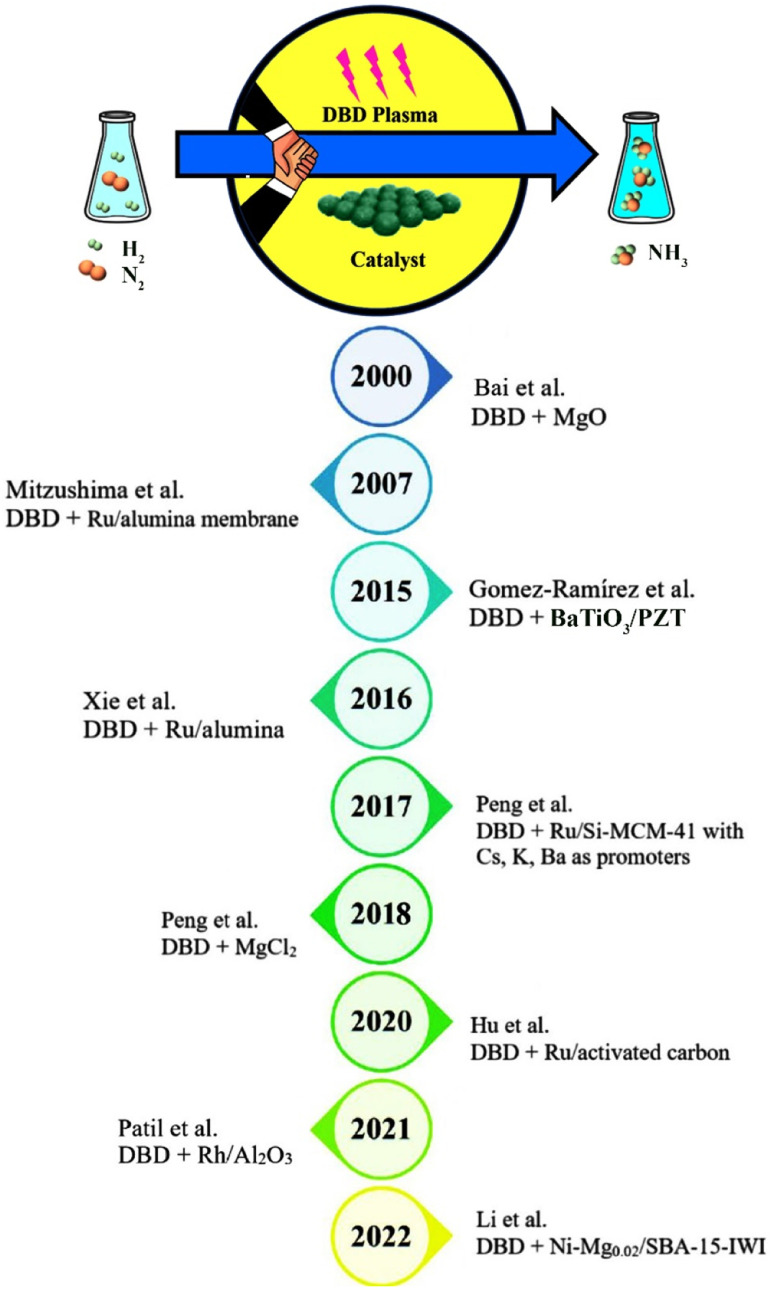
A summary of developments of plasma-catalysis ammonia synthesis in DBD plasma reactors along with various catalysts since 2000.

Researchers have investigated ammonia synthesis using plasma-assisted catalysis in a variety of reactor configurations and operating conditions with a broad range of catalysts.^52^ Ammonia has been synthesized using different types of discharges including glow discharge,^53^ RF and microwave discharges,^54–59^ arc discharge,^60^ and DBD up to now. Surprisingly, the majority of the studies on the plasma ammonia synthesis from N_2_ and H_2_ have been carried out using a DBD plasma at atmospheric pressure and mild temperatures. As a matter of fact, ammonia yield is enhanced when plasma is coupled with a catalyst, but some literature has reported production of ammonia without a catalyst. Accordingly, Kubota *et al.* synthesized ammonia without the use of a catalyst in a plasma–liquid system in 2010.^61^ While many attempts have been made to produce ammonia in DBD reactors since the past several decades, plasma-catalytic ammonia synthesis in these reactors has been extensively explored since 2000.^62^ A brief summary of developments of plasma-catalysis ammonia synthesis in DBD plasma reactors since 2000 is shown in Fig. 5.

In order to synthesize ammonia in the plasma-catalysis system, two main factors must be taken into account: the catalyst and the plasma parameters, which will be discussed further in this section.

### 3.1. Catalyst development

The development of catalysts plays a critical role in improving NTP ammonia synthesis.

In the ammonia synthetic process, several different types of materials as catalysts in connection with plasma have been studied to date, according to literature reports. Among these, oxides and supported oxides,^63–65^ zeolites,^66–68^ as well as metals and supported metals^69–74^ are the most common catalysts.

### 3.2. Oxides and supported oxides

In addition to TiO_2_, MgO, CaO, quartz wool, and BaTiO_3_ as catalyst supports,^65^ alumina as one of the most frequent catalysts in this group is used in the plasma-catalytic synthesis of ammonia. Xie *et al.* reported NH_3_ synthesis process from N_2_ and H_2_ over the Al_2_O_3_ catalyst in a dielectric barrier discharge plasma reactor.^63^ They found that ammonia produced using Al_2_O_3_ was more than that produced without it. Furthermore, it was stated that the presence of alumina resulted in higher ammonia production in this plasma process due to its certain catalytic activity. In another study, Zhu *et al.* synthesized ammonia from N_2_ and H_2_ by using acidic γ-Al_2_O_3_, alkaline γ-Al_2_O_3_ and neutral alumina pellets in a dielectric barrier discharge plasma reactor.^64^ The results demonstrated that the plasma-catalytic synthesis of ammonia increased in the presence of all types of γ-Al_2_O_3_ from 15.6% to 44.4%, notably the alkaline γ-Al_2_O_3_, in comparison with the plasma reaction without packing materials. This implies that the attendance of a packing material such as oxides can affect both the discharge power required to ignite the plasma and the plasma discharge characteristics.^62^

### 3.3. Zeolites

Another group of catalysts in the ammonia synthesis process using plasma-assisted catalysis is zeolites. Gorky *et al.* examined atmospheric-pressure nonthermal plasma synthesis of ammonia over zeolitic imidazolate frameworks (ZIFs) in a DBD reactor.^66^ Based on the results obtained from this study, the dipole–dipole interactions between the polar ammonia molecules and the polar walls of the aforementioned ZIFs caused relatively low ammonia uptakes, low storage capacity, and eventually high observed ammonia synthesis rates. Shah *et al.* also found that the use of zeolite 5A for the plasma-catalytic synthesis of ammonia led to an increased catalytic performance.^67^ Alternatively, an energy yield of 15.5 g-NH_3_ per kW per h was obtained with zeolite 5A at an equimolar N_2_/H_2_ ratio, which is at least 50 times higher than that without zeolite. Hence, the presence of the zeolites as active catalysts in the DBD reactor can promote the ammonia yield and even energy yield so that ammonia yield of 5.31% was obtained in the presence of zeolite beta.^68^

### 3.4. Metals and supported metals

The use of metals and supported metals as catalysts, especially transition metals, for ammonia production in the plasma system has been extensively studied by researchers.^69–74^ Hu *et al.* investigated the synthesis of NH_3_ on activated carbon-supported metal (Ru, Co, Ni, and Fe) catalysts in a coaxial dielectric barrier discharge reactor.^71^ Based on the reported results, the highest ammonia concentration of 3026.5 ppm and energy efficiency of 0.72 g kW h^−1^ were obtained with Ru/AC. These results indicate that coupling the dielectric barrier discharge with an activated carbon support increased the NH_3_ concentration by 11.0–22.5% compared to plasma alone. Moreover, the synthesis of NH_3_ was increased by up to 37.3% by doping active metal on activated carbon. Li *et al.* found that the presence of the Ni/LaOF catalyst with dual active centers in a dielectric barrier discharge system can be efficient on the ammonia synthesis rate.^72^ Accordingly, the ammonia synthesis rate in the presence of Ni/LaOF was about two times higher than when pure LaOF was used and at least 30 times higher than when plasma was used alone. In another study, plasma catalytic synthesis of NH_3_ on Al_2_O_3_ supported transition metals such as Co, Ni, Co–Ni was carried out in a DBD plasma reactor.^73^ It was found that the highest NH_3_ synthesis rate in this study was achieved with Co–Ni/Al_2_O_3_. In this case, this bimetallic catalyst is not only cheaper, but also reduces the acidity of the catalyst surface and increases the plasma discharge, which benefits the ammonia synthesis. In addition to the catalysts described above, other catalysts for plasma catalytic ammonia synthesis have been proposed by various research groups. For example, Iwamoto *et al.* tested wool-like electrodes for ammonia synthesis in a DBD reactor.^75^ Among these catalysts studied, Au showed the highest catalytic activity. Another catalyst reported in NH_3_ production is a tubular membrane-like catalyst.^76,77^ The presence of metals such as Ru, Pt, Ni, and Fe on the alumina led to an increase in ammonia synthesis by enhancing the hydrogenation of N(a) species (species adsorbed on an adsorbent are prefixed with “(a)”).^77^ A number of catalysts used in plasma catalytic ammonia synthesis are listed in Table 1.

**Table 1:** Summary of literature on the plasma ammonia synthesis using various catalysts in dielectric barrier discharge (DBD) reactors

Entry	Catalyst	H_2_/N_2_ rate	Feet flow rate (ml min^−1^)	Voltage (kV)	Power (W)	NH_3_ yield (%)	Energy yield (g kW^−1^ h^−1^)	Energy cost (MJ mol^−1^)	Year	Ref.
1	Pd wound on electrode	3	Batch		12.5	3.13			1969	78
2	MgO smeared on electrode	0.8	2266.7	0.58		0.33			2000	79
3	Ru/alumina membrane	3	40	4.5	127	4.36	0.37	163.9	2004	76
4	Ru/alumina membrane	3	30	4.5	127	4.62	0.4	154.7	2007	77
5	BaTio_3_/PZT	1	38.3	3		2.8	0.9	136	2015	80
6	Alumina and DLC coated alumina	3	60	15	90.5	0.67	0.18	340	2016	81
7	Cs–Ru/MgO	3	4000	6		2.41	2.3	26.6	2016	82
8	Cu wool	1	100	5		3.5	3.3	18.5	2016	83
9	Ru/alumina	1.5					6.4	95.6	2016	84
10	Ni/silica with barium titanate as dielectric	3	25	20	107	12	0.75	81	2017	85
11	Lead zirconium titanate	3	11.5	5.5		0.5	0.75	81.6	2017	86
12	Ru–Mg/alumina	4	2000	5.4		2.55	35.7	1.7	2017	87
13	Ru/alumina	3	1000	7.5		0.05	1.9	32.2	2017	65
14	Ru/Si-MCM-41 with Cs, K, Ba as promoters	1		5			1.7	3.6	2017	88
15	Au wool	1	100		128.7		0.58		2017	75
16	Ni nanoparticles/alumina	2	100		10	2	0.89	68.9	2018	89
17	MgCl_2_	1	4000	6.4			20.5	2.9	2018	90
18	Ni/Al_2_O_3_	2	56	12	25.1		0.56		2019	91
19	Ru/α-Al_2_O_3_	0.5	120	8	38.4	1.49	1.89	32.39	2019	92
20	Zeolite 5A	1	25	7.5	13.3		15.5	3.95	2020	67
21	Alkaline γ-Al_2_O_3_	3	100		24.25		6.58	9.30	2020	64
22	Ru/activated carbon	3	100		13.3		0.72	85	2020	71
23	Rh/γ-Al_2_O_3_	0.5	100	8	24	1.43	0.94	65	2021	93
24	Co–Ni/Al_2_O_3_	1	200		30.81		0.83		2022	73
25	Ni–Mg_0.02_/SBA-15-IWI	1	20	9.5			1.05		2022	94

### 3.5. Investigation of the plasma parameters

**Fig. 6:**
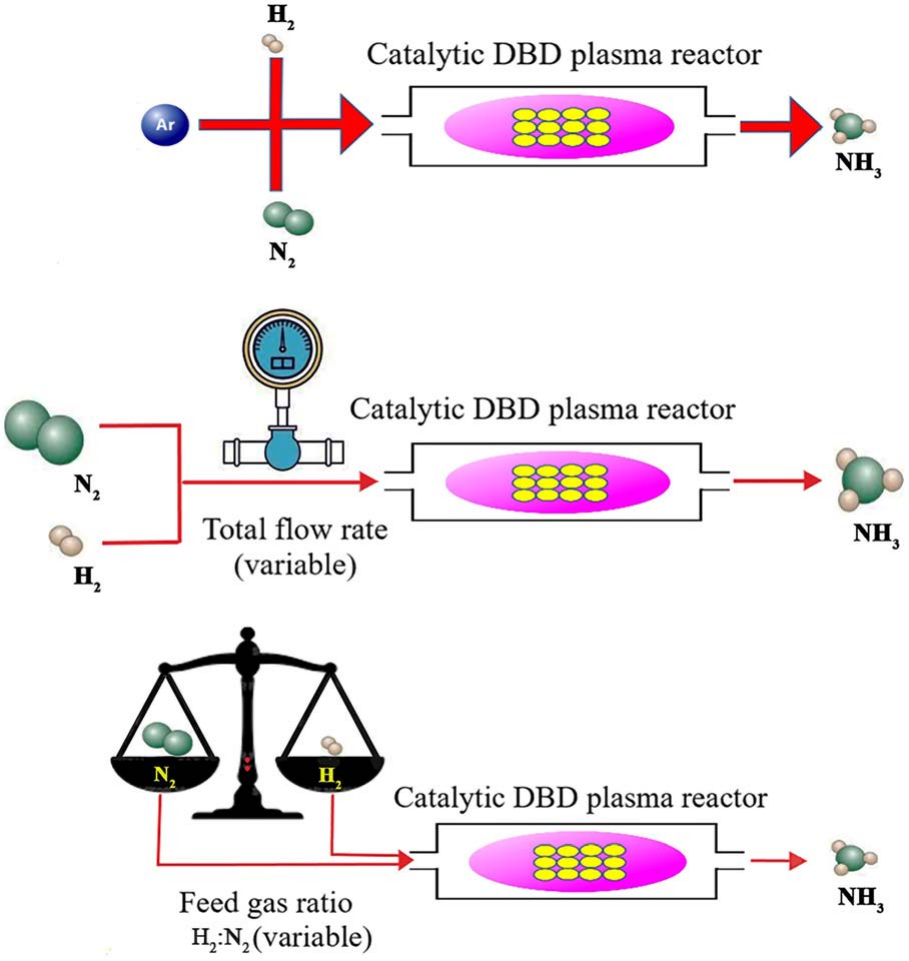
Some effective plasma parameters in the plasma-catalytic synthesis of ammonia, including the argon addition, the flow rate of reactants, and the feed gas ratio.

Apart from the significance of catalysts in plasma-assisted catalysis synthesis of ammonia, the effect of plasma parameters should also be considered, some of which, including the argon addition, the flow rate of reactants, and the feed gas ratio, are discussed below (Fig. 6).

### 3.6. Effect of argon addition

Argon can lead to a change in ammonia production during the plasma catalytic synthesis of ammonia from N_2_ and H_2_. Indeed, the addition of argon led to an increase in the production of N_2_^+^ through charge transfer between Ar^+^ and N_2_, enhancing the formation of the NH radical as an intermediate of ammonia, as shown in eqn (1)–(3).^95,96^

1 N_2_ + Ar^+^ → N_2_^+^ + Ar

2 N_2_^+^ + H_2_ → N_2_H^+^ + H

3 N_2_H^+^ + e^−^ → NH + N

It was realized that when argon was introduced, nitrogen and hydrogen conversion was improved, and this improvement was more pronounced when argon content was higher.^92^ Although this improvement came at a cost of production rate and energy consumption, the actual value of N_2_ and H_2_ was reduced with an increase in the concentration of argon. Accordingly, it was reported that the conversion of reactants improved more than five times in a catalytic DBD reactor with 87% argon dilution, with a 1.5 times rise in energy consumption and a 31% decrease in NH_3_ generation compared to no dilution condition. In this study, the addition of argon appears to be able to affect the conversion of reactants in a catalytic dielectric barrier discharge reactor more than in a DBD reactor without a catalyst.

### 3.7. Effect of flow rate

The influence of flowrate of reactants on the plasma-catalytic synthesis of ammonia has been examined by several research groups.^69,73,97,98^ The increase in the flow rate of gas can lead to a decrease in the residence time of reactive species in the plasma system.^69^ Additionally, as the gas flow rate increases when the pressure remains constant, more raw reactant gas is added to the system, increasing the chances of reactive particles colliding, which is beneficial to ammonia production.^73^ Hence, the reaction gas flow rate affects the ammonia production rate. To assess the effect of flow rate on the ammonia production rate, the DBD reactor packed with the Al_2_O_3_ supported transition metals such as Co, Ni, and Co–Ni was tested for several gas flow rates. The results indicated that increasing the total gas flow rate as a plasma parameter can improve the synthesis rate of ammonia, although the NH_3_ production growth rate reduced at a flow rate greater than 120 ml min^−1^. Ma *et al.* also investigated the effect of total gas flow rate on NH_3_ synthesis and energy cost under ambient conditions using the tangled Cu electrode at a constant molar ratio of N_2_/H_2_ of 1 : 1 and a discharge power of 20 W.^97^ It was found that the ammonia concentration decreased with increasing total flow rate as the number of collisions between reactant molecules and energetic electrons and other reactive species decreased. It has also been reported that when the total flow rate increased from 50 to 250 ml min^−1^, the energy cost of NH_3_ production reduced from 139.3 to 59.0 MJ mol^−1^.

As a matter of fact, the higher flow rate, however, enhances the total number of reactants passing through the plasma zone and promotes the conversion of molecules at a constant discharge power.

### 3.8. Effect of feed gas ratio

**Fig. 7:**
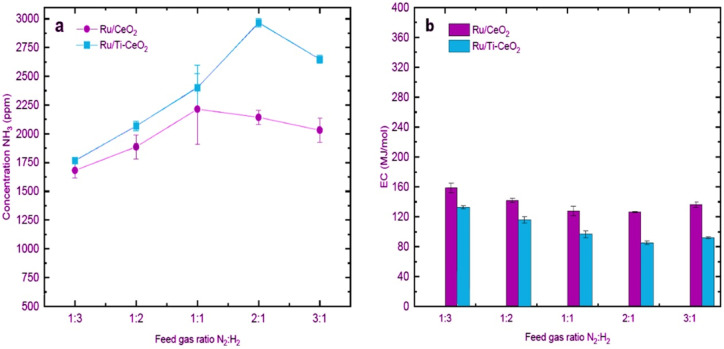
Effect of different feed gas ratios on (a) NH_3_ concentrations and (b) ECs. Reproduced from ref. 70 with permission from Elsevier B.V., copyright 2023.

H_2_ : N_2_ gas ratios in plasma catalytic NH_3_ synthesis can affect the concentration and production rate of ammonia as well as the energy consumption of the plasma system.^99^ To investigate the effect of feed gas ratio on NH_3_ synthesis, van Raak *et al.* obtained the ammonia concentrations as well as energy consumption for the different feed gas ratios on Ru/CeO_2_ and Ru/Ti–CeO_2_ in a coaxial DBD reactor (Fig. 7).^70^ As can be seen, the highest concentration for Ru/CeO_2_ was 2215 ppm at a N_2_ : H_2_ ratio of 1 : 1, while the maximum concentration for Ru/Ti–CeO_2_ was 2965 ppm at a ratio of 2 : 1. This result illustrated that as N_2_ increases, the difference between the two catalysts becomes more significant. On the other hand, the lowest energy consumption (ECs), namely 85.4 MJ mol^−1^, was obtained for Ru/Ti–CeO_2_ at a ratio of N_2_ : H_2_ = 2 : 1. At the same ratio, the minimum energy consumption for Ru/CeO_2_ was reported to be 126.5 MJ mol^−1^. This implies that N_2_-rich environments combined with Ru-catalysts led to the minimum ECs.

### 3.9. Reaction mechanisms in plasma-catalysis ammonia synthesis

Optical emission spectroscopy (OES) is more commonly utilized than other analytical techniques to determine the plasma species that were obtained from ammonia in a catalytic DBD reactor. Nevertheless, OES is not able to detect all species, including N*.^100^ The results of optical emission spectroscopy to identify excited species in the N_2_–H_2_ plasma reaction using the DBD plasma in the presence of various catalysts have been reported by several research groups, as shown in Table 2.^80,91,93,100–102^ Although the mechanisms of ammonia synthesis in a non-thermal plasma catalysis system have been reported and described by several groups,^103–109^ Hong *et al.* have presented for the first time a detailed kinetic modelling of non-equilibrium N_2_–H_2_ atmospheric pressure discharges for catalytic NH_3_ synthesis.^110^ As we all know, to form ammonia in the gas phase, the bonds of both molecular hydrogen and molecular nitrogen must be broken.^62^ This can be achieved *via* their collision with high-energy electrons in the plasma, as shown in eqn (4) and (5).^93^ Thus, when N_2_ molecules collide with high-energy electrons, it is possible for N_2_ molecules to excite, ionize, and even dissociate. The excited N_2_ species undergo parallel reactions either by homogeneous reaction with molecules of H_2_ or by heterogeneous reaction with molecules of H_2_ adsorbing on the catalyst surface, as shown in eqn (6) and (7). In both cases, the NH_*x*_ molecules formed are capable of reacting with H_2_ to produce NH_3_ molecules, as shown in eqn (8). Catalysts can assist in the adsorption of NH_3_ onto their surfaces and, if the reaction temperature is above about 250–300 °C, the molecules can thermally decompose, as shown in eqn (9).4
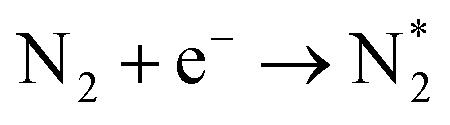
5
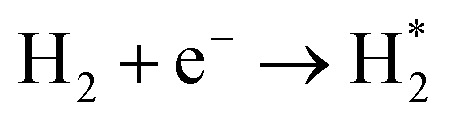
6
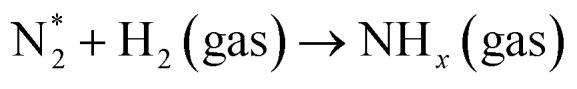
7
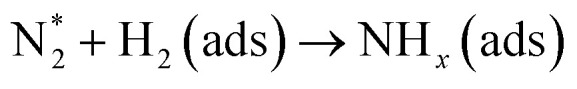

8 NH_*x*_ + H_2_ → NH_3_

9 If *T* > 300 °C: NH_3_ → N_2_ + H_2_

In another study, Mizushima *et al.* proposed a mechanism for the formation of ammonia in the plasma system.^77^ In this reaction pathway, 
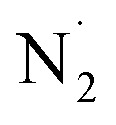
 obtained from N_2_ plasma reaction can form atomic N(a) species. The N(a) atoms react with H atoms or activated 
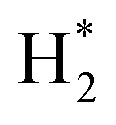
 molecules to form NH_3_. They stated that the presence of metals on alumina can accelerate the hydrogenation of N(a) species, increasing NH_3_ yields. Based on these results, it is concluded that metals can act as catalysts in the formation of ammonia by N_2_–H_2_ plasma.

**Table 2:** Some species detected by OES under various operating conditions of the dielectric barrier discharge plasma reactor

Entry	Species	Electronic transition	Band	Wavelength (nm)	Year	Ref.
1	N_2_ (SPS)	C^3^Π → B^3^Π	0–0	357.9	2015	80
2	N_2_^+^ (FNS)	B^2^Σ^+^_u_ → X^2^Σ^+^_g_		391.4	2015	80
3	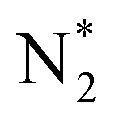		0–0	337	2015	80
4	NH*	A^3^Π → X^3^∑	0–0	336	2015, 2021, 2022	80, 93 and 101
5	N^2+^			<250	2021	93
6	N			742, 818.5, 822, 824, 939, and 1011	2022	102
7	H_α_			656.3	2019, 2022, 2022	91, 101 and 102
8	H_β_			486.2	2022	102

In addition to OES, the electron impact molecular-beam mass spectrometer (EI-MBMS) as a powerful approach was employed for the detection of gas-phase radicals and molecules in the plasma processes even at atmospheric pressure. Recently, Zhao *et al.* used an EI-MBMS for *in situ* identification of gas-phase species in a dielectric barrier discharge plasma-assisted catalytic reactor.^111^ They succeeded for the first time to identify N_2_H_2_, NNH, and NH as the gas-phase species in plasma-assisted NH_3_ synthesis. Gas-phase NNH can be produced by the following eqn (10) and (11):

10 N(g) + H(g) ⇄ NNH(g)

11 N(v) + H(g) ⇄ NNH(g)

Moreover, according to the following eqn (12) and (13), N_2_H_2_ was obtained from the reaction of NNH(g) with H(g) or H_2_(g).

12 NNH(g) + H(g) ⇄ N_2_H_2_(g)

13 NNH(g) + H_2_(g) ⇄ N_2_H_2_(g) + H(g)

Based on the observations made in this study, it was determined that NNH and N_2_H_2_ species are important for the production of ammonia in a dielectric barrier discharge reactor because of their interactions. The reaction of N(s) and NH(s) as the surface formed intermediates with H(g) and H(s) can lead to the production of NH_2_(s) and eventually NH_3_(s), as shown by the following eqn (14)–(19)

14 N(s) + H(g) ⇄ NH(s)

15 N(s) + H(s) ⇄ NH(s) + s

16 NH(s) + H(g) ⇄ NH_2_(s)

17 NH(s) +H(s) ⇄ NH_2_(s) + s

18 NH_2_(s) + H(g) ⇄ NH_3_(s)

19 NH_2_(s) +H(s) ⇄ NH_3_(s) + s

## 4. Conclusion

Major efforts have been undertaken to develop an alternative and environmentally friendly technology for the production of ammonia under the mild conditions. Plasma catalysis is a promising option for the ammonia production at atmospheric pressures and temperatures close to ambient. In addition to this, the plasma-catalytic has the significant potential to resolve the crises of ammonia synthesis present in the Haber–Bosch process such as the consumption of fossil fuels and environmental pollution. Although currently, ammonia production quantities achievable by plasma reactors are not comparable to those achievable in large Haber–Bosch reactors, by optimizing the catalyst and DBD reactor and studying the kinetics and reactant composition, it is anticipated that plasma technology, particularly DBD plasma, due to its ability to easily create nonequilibrium conditions, will be able to significantly improve the production of NH_3_. This review focused on the plasma synthesis of NH_3_ in the DBD reactor packed with different catalysts. In summary, dielectric barrier discharge plasma combined with catalysts can increase not only the ammonia yield but also the synthesis rate. As was mentioned, a wide range of catalysts was used in the plasma-assisted NH_3_ synthesis process and their effect was examined on ammonia yield and the energy yield. Consequently, reasonably high yields of ammonia in many experiments were reported, but energy efficiencies were not satisfactory. In addition to the effect of the catalyst, other process parameters such as argon addition, the flow rate, and the feed gas ratio would have a pronounced influence on the NH_3_ synthesis. Recently, the effects of process parameters on NH_3_ concentration and energy efficiency have been systematically investigated using the central composite design model and response surface methodology (CCD-RSM).^69^ Based on the analysis of variance (AVONA), the most important variables affecting the NH_3_ concentration and energy efficiency of the plasma-assisted NH_3_ synthesis process were the plasma discharge power and the gas flow rate, respectively. Considering the available results of the experiments conducted so far and the proposed mechanisms for ammonia synthesis in the plasma catalyst system, it is concluded that further research is needed to optimize the plasma-catalysis NH_3_ synthesis process. Therefore, it is expected that the number of experimental and modelling research will increase in the future. However, the selection of efficient catalysts or the innovation of new catalysts as well as the proper design of the DBD reactor in the catalytic plasma system can have a significant impact on ammonia production, energy efficiency and even the production of by-products.

## Conflicts of interest

The author declares no known competing interests.

## Supplementary Material
